# Floating Atheromatous Plaque in the Common Femoral Artery: A Rare Case With Implications for Diagnosis and Management

**DOI:** 10.7759/cureus.117624

**Published:** 2026-10-08

**Authors:** Julian M Paione Oleszuk, Mohammad Ghasemi, Jens Achenbach, Clara Edith Engel, Zoran Rancic

**Affiliations:** 1 Vascular Center, Lachen Hospital, University Hospital Basel, Basel, CHE; 2 Vascular Center, Lachen Hospital, Lachen, CHE; 3 Department of Surgery, Lachen Hospital, Lachen, CHE; 4 Vascular Center, Lachen Hospital, University of Zurich, Zürich, CHE

**Keywords:** atherosclerosis, calcified plaque, common femoral artery, duplex ultrasound, femoral endarterectomy, floating plaque, peripheral arterial disease

## Abstract

Floating plaques are well recognized in the carotid artery but are rarely described in the femoral circulation. We present the case of a 73-year-old man with advanced peripheral arterial disease and features of chronic limb-threatening ischemia, including chronic left foot ulceration with a non-healing second-toe ulcer with exposed bone, in the background of prior transmetatarsal amputations and delayed wound healing. A mobile plaque was identified in the left common femoral artery on dynamic B-mode ultrasound, whereas computed tomography angiography showed eccentric calcified stenosis without a definite floating component. The patient underwent femoral endarterectomy with patch repair, and histology confirmed a ruptured calcified atheromatous plaque. Recovery was uneventful, and 12-month follow-up showed no restenosis. This case highlights the diagnostic value of dynamic B-mode ultrasound for detecting mobile femoral atheromatous lesions.

## Introduction

Atherosclerotic lesions range from early intimal thickening to advanced forms, with variable clinical relevance [1]. Among these, the floating atheromatous plaque is well described in the carotid artery but is rarely reported in the femoral circulation [2,3]. This underreported presentation may be overlooked in routine assessment, and most available evidence is extrapolated from other vascular territories.

Floating atheromatous plaques may result from plaque rupture or ulceration, with a mobile plaque component protruding into the arterial lumen. Other mobile intraluminal structures may include an intimal flap or thrombus adherent to an atherosclerotic plaque. Importantly, a floating atheromatous plaque consists primarily of atherosclerotic tissue, whereas a thrombus represents a blood-derived intraluminal mass that may form secondarily on a disrupted plaque [2,4]. While their clinical relevance in carotid circulation is well recognized because of the risk of embolic stroke, in peripheral arteries they may similarly be associated with thromboembolism, thrombosis, or occlusion, potentially contributing to ischemic complications [2,5,6]. Limited published data in the femoral territory make it difficult to characterize their true frequency and clinical impact, particularly in hemodynamically distinct regions such as the common femoral artery (CFA).

Dynamic B-mode ultrasound enables direct visualization of mobile plaque components, whereas computed tomography (CT) angiography and magnetic resonance imaging (MRI) may fail to capture subtle motion and may depict only static features such as eccentric calcification or ulceration [7,8]. In very delicate mobile flaps, color duplex may even obscure the finding, making B-mode video imaging particularly valuable for diagnosis [9].

Because femoral floating plaques are rarely described, management strategies are largely guided by general principles applied to unstable atherosclerotic lesions in other vascular beds [10,11]. Given the potential risk of thromboembolic events, surgical removal with common femoral endarterectomy and patch repair represents a reasonable approach in selected patients.

We report a rare case of a floating atheromatous plaque in the left CFA, identified by dynamic B-mode ultrasound and successfully treated with surgical endarterectomy.

## Case presentation

A 73-year-old man with severe peripheral arterial disease presented with chronic left foot ulceration and a history of delayed wound healing, leading to prior transmetatarsal amputations, negative-pressure wound therapy, and repeated debridements. His medical history included chronic venous insufficiency. Major atherosclerotic risk factors were arterial hypertension, dyslipidemia with suboptimal low-density lipoprotein levels despite atorvastatin therapy, and long-standing heavy alcohol consumption.

On examination, he had a non-healing ulcer on the second toe and absent distal pulses on the left. The contralateral limb had no rest pain or limiting claudication. Physiologic testing showed an ankle-brachial index of approximately 0.45 on the left (toe pressure ~38 mmHg), consistent with moderate ischemia. Based on wound depth and perfusion parameters, the estimated Society for Vascular Surgery WIfI classification was W3-I2-fI0 (wound grade 3, ischemia grade 2, foot infection grade 0).

Dynamic B-mode ultrasound demonstrated an oscillating plaque in the left CFA with an estimated 50%-60% stenosis, consistent with a floating atheromatous plaque (Figure 1; Video 1). The profunda femoris was patent, with multiphasic flow in the superficial femoral, popliteal, and anterior tibial arteries; the posterior tibial artery was occluded, while the peroneal artery showed biphasic flow.

**Figure 1:**
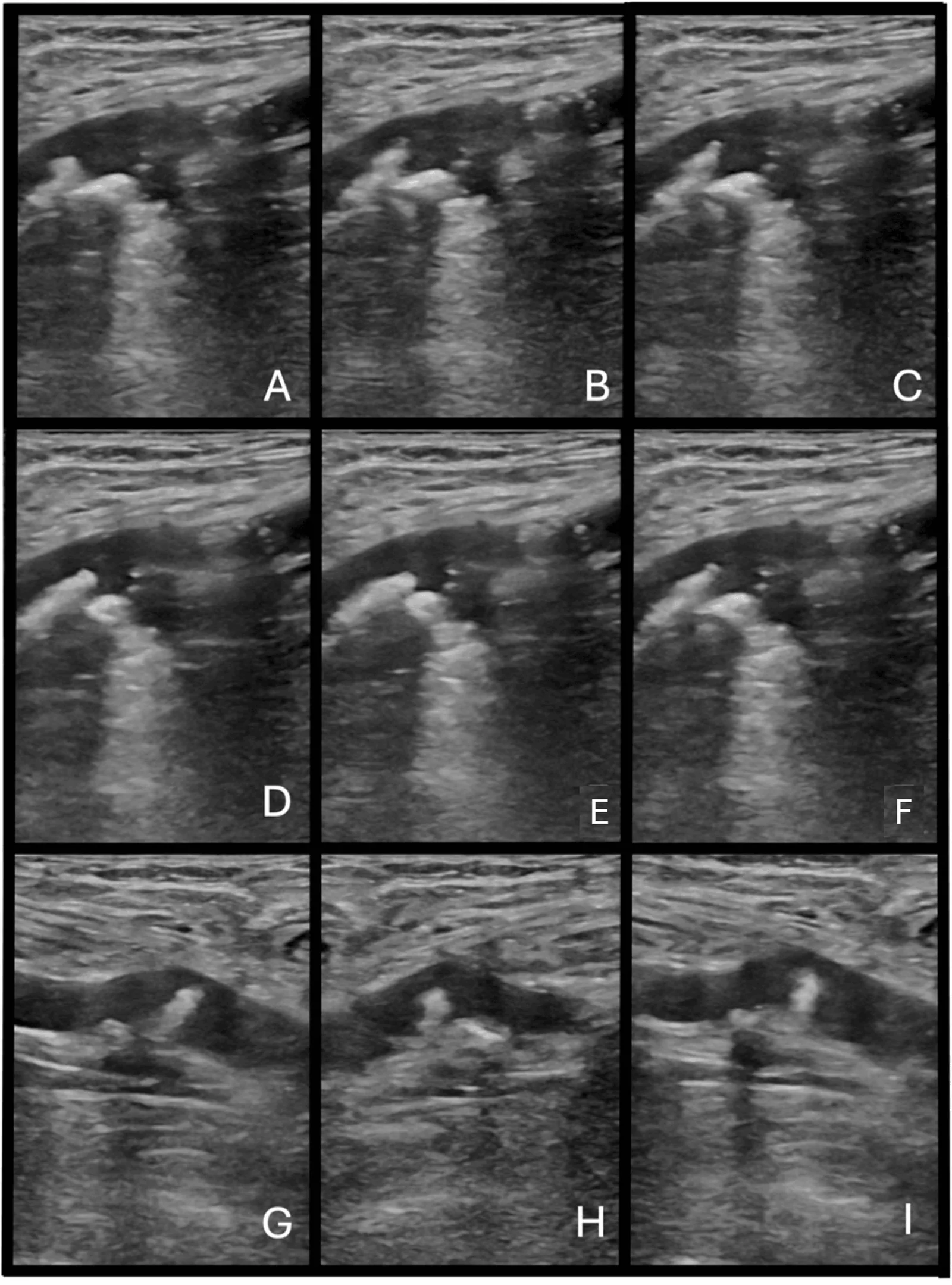
Sequential B-mode ultrasound images demonstrating a floating common femoral artery plaque. Sequential B-mode ultrasound images of the common femoral artery demonstrating a mobile, floating atheromatous plaque (panels A–I). The plaque oscillates within the arterial lumen across frames, confirming its dynamic nature. Dynamic B-mode imaging provides direct visualization of lesion instability and potential embolic risk.

**Video 1:** Dynamic B-mode ultrasound of a mobile femoral plaque (video sequence).

CT angiography demonstrated stenosis of the left CFA with multiple eccentric calcified plaques. The mobile component identified on ultrasound was not visualized, and interpretation was further limited by artifact from the patient’s hip prosthesis, reducing the reliability of CT imaging in this region (Figure 2).

**Figure 2:**
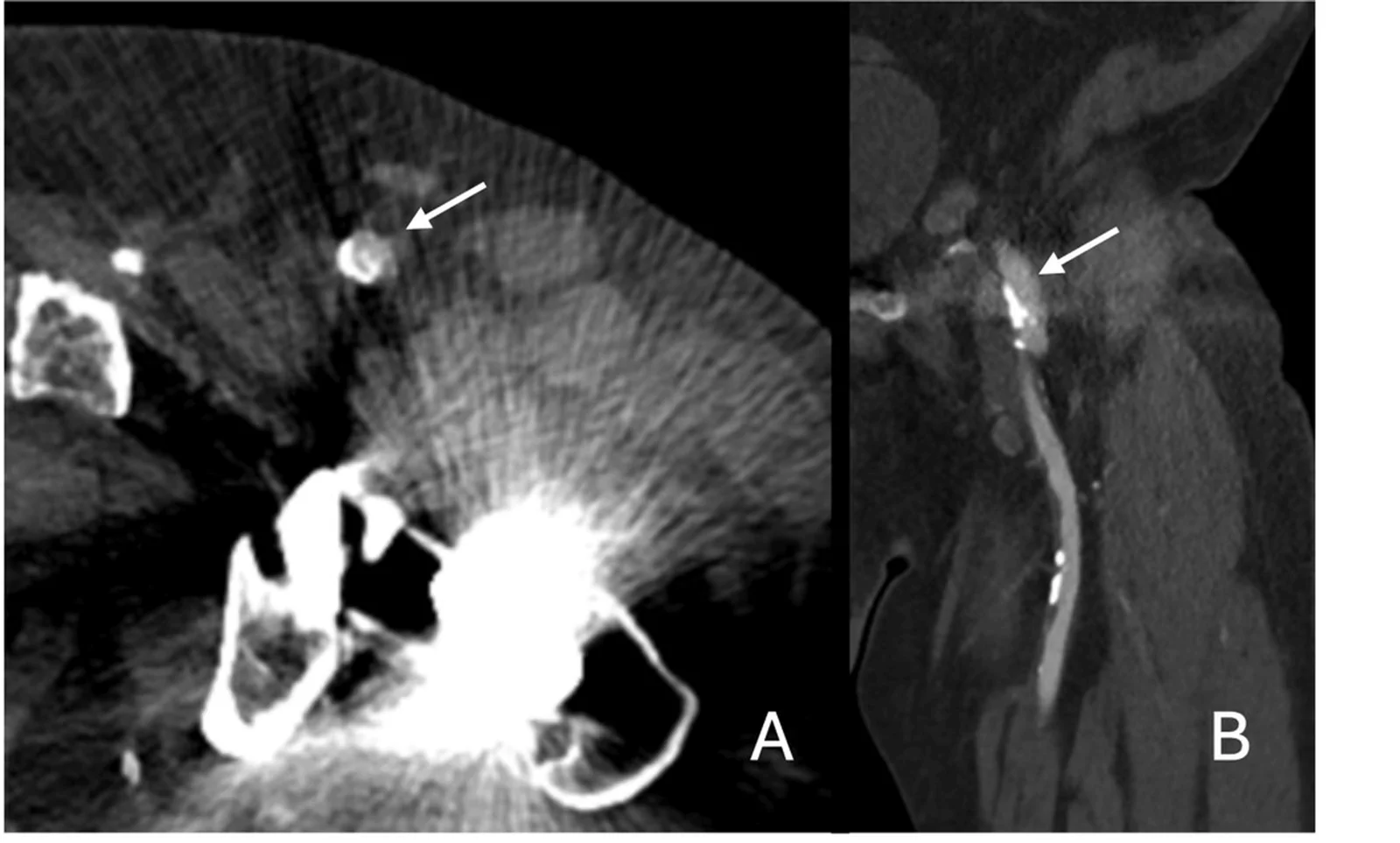
Computed tomography (CT) angiography demonstrating common femoral artery (CFA) plaque and stenosis. (A) Axial CT slice of the pelvis showing eccentric calcified plaque in the CFA. (B) Coronal CT reconstruction of the lower extremity demonstrating segmental CFA stenosis with adjacent hip prosthesis-related imaging artifact. The mobile (floating) component was not visualized on CT. The arrow indicates the atherosclerotic plaque.

Given the mobile plaque component, CFA stenosis, chronic tissue loss, and potential embolic risk, femoral endarterectomy was performed under general anesthesia without complications. The procedure included removal of the plaque (Figure 3) and arterial reconstruction using an Invengenx® bovine pericardial tissue patch (Tisgenx, Inc., Irvine, CA, USA). Intraoperative Doppler confirmed restoration of blood flow.

**Figure 3:**
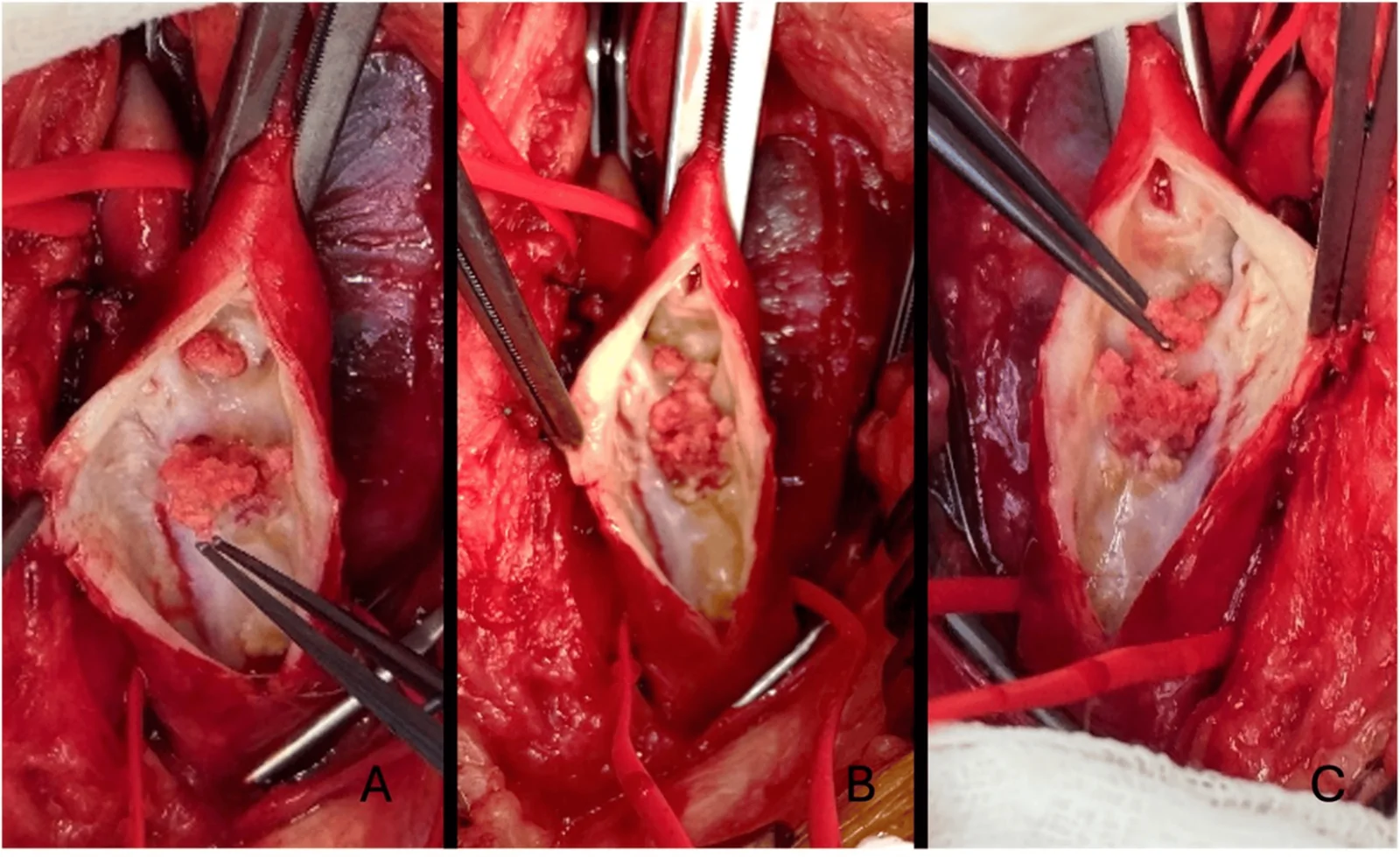
Intraoperative common femoral artery (CFA) endarterectomy showing the floating plaque component. (A–C) Intraoperative photographs during left CFA endarterectomy. (A) Arteriotomy of the CFA with an atheromatous plaque visible within the lumen. (B) Identification of a mobile, floating plaque component protruding into the lumen. (C) Removal of the plaque/fragment demonstrating its irregular, ulcerated surface.

The excised plaque was sent for histological analysis, which showed arterial wall fragments with pronounced coarse calcification and ruptured atherosclerosis, with a small amount of adherent fibrin and no lipid identified in the examined specimen (Figure 4). These findings are consistent with an advanced ruptured atheromatous plaque, supporting the dynamic appearance observed on ultrasound.

**Figure 4:**
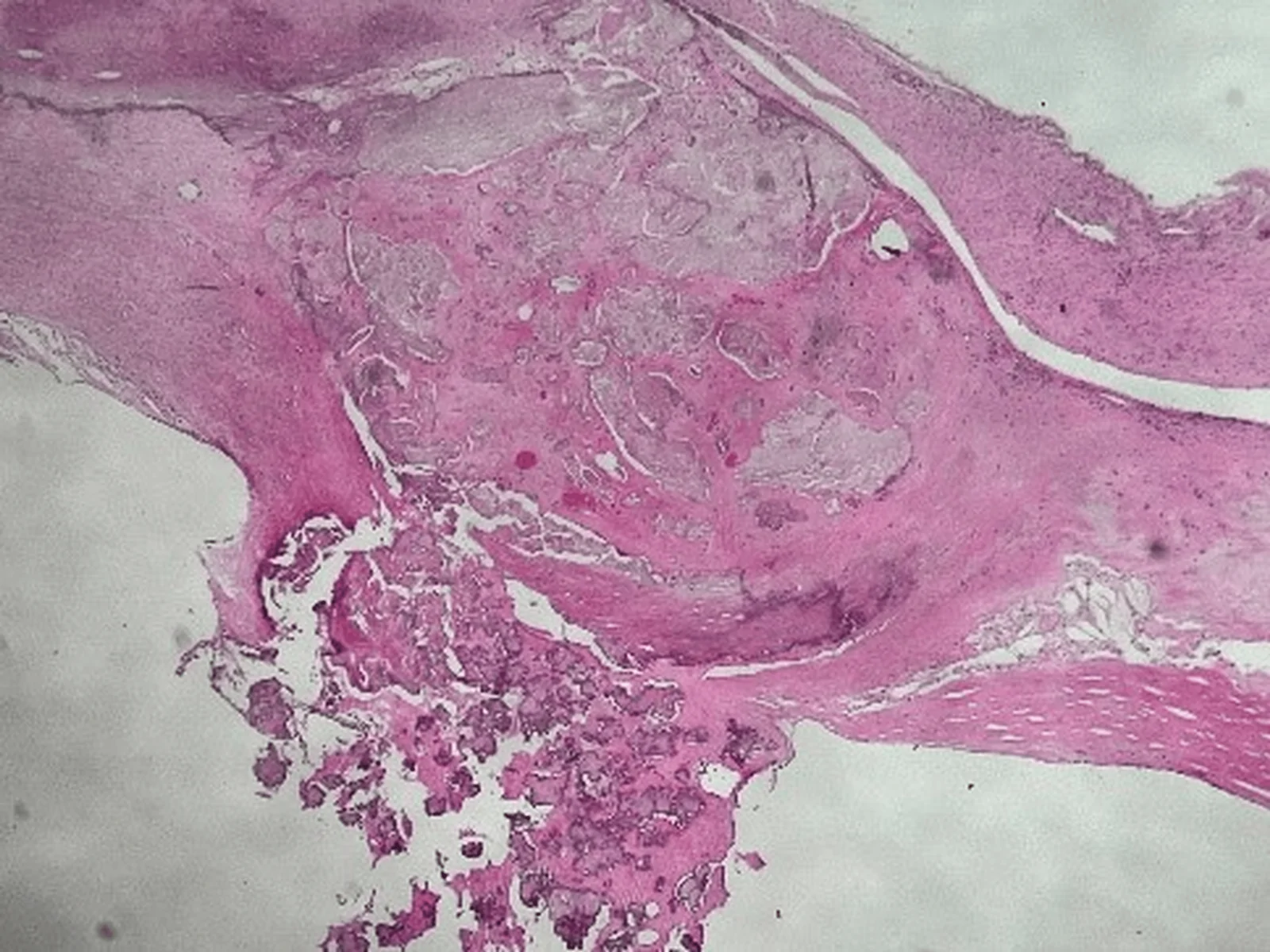
Histopathology of the excised common femoral artery (CFA) plaque. Histologic section of the excised left CFA plaque showing an advanced atherosclerotic lesion with marked calcification and surface disruption/rupture, consistent with an unstable plaque.

The postoperative course was uneventful. The patient was discharged on the fourth postoperative day on acetylsalicylic acid 100 mg once daily and rivaroxaban 2.5 mg twice daily. At 12 months, the patient reported no new ischemic symptoms. Follow-up duplex ultrasonography demonstrated no evidence of restenosis at the endarterectomy site. Physiologic testing showed stable perfusion parameters compared with preoperative values. The persistent non-healing ulcer of the left second toe ultimately required transphalangeal amputation.

## Discussion

Floating intraluminal masses have been described in multiple arterial territories, where they are frequently thrombotic in nature [12,13]. Histologic analyses of carotid floating plaques suggest that many lesions are ulcerated and highly unstable, sometimes with limited superimposed thrombus [14]. Whether femoral floating plaques share the same histomorphologic characteristics remains uncertain, as published data for the femoral territory are scarce. Our report adds to the limited literature by describing a rare floating atheromatous plaque in the CFA. Even when reported less often than carotid lesions, the clinical consequences of plaque instability, i.e., embolization, thrombosis, and progression to occlusion, are relevant across vascular beds and justify careful assessment and timely management.

Femoral lesions may differ from carotid plaques in composition and are often more calcified, which can reduce the expected benefit of medical therapy alone and make surgical removal a reasonable option in selected patients [3,15]. From a guideline standpoint, optimized best medical therapy remains the foundation of peripheral artery disease management [10].

From a pathophysiologic standpoint, a mobile intraluminal structure can represent an ulcerated atherosclerotic plaque with a mobile component, an intimal flap/dissection, or an associated thrombus. Exposure of subendothelial collagen and tissue factor after plaque rupture may promote thrombus formation and increase ischemic risk [4]. Importantly, beyond the degree of stenosis, plaque stability is a critical determinant of clinical risk. A floating component, whether plaque material or superimposed thrombus, suggests instability and potential embolic risk, supporting prompt diagnosis and consideration of intervention. In our case, the patient was already receiving best medical therapy [16].

Diagnosis is typically established using noninvasive imaging. Dynamic B-mode ultrasound is particularly valuable for detecting floating plaque components because it allows real-time visualization of mobility [16]. Nevertheless, B-mode ultrasound is operator-dependent, and its diagnostic accuracy may vary according to the experience of the examiner. CT angiography can demonstrate calcification and ulceration but lacks temporal resolution to capture oscillating components. Angiography may be useful to delineate lesion extent and morphology, support procedural planning, and detect additional stenoses or occlusions that may not be evident on ultrasound alone.

Peripheral arterial disease is a major cause of lower-limb amputation, and ischemic complications remain a key contributor to morbidity and mortality, underscoring the importance of early recognition of unstable atherosclerotic lesions and timely intervention when indicated [10]. A floating plaque may develop after rupture or ulceration of an atherosclerotic lesion, where a mobile component protrudes into the lumen and moves with pulsatile flow. Depending on composition, these mobile elements may be fibrotic and/or calcified, with variable degrees of adherent thrombus. In contrast, a free-floating thrombus is a fresh, soft thrombotic mass that moves within the bloodstream and may arise from stasis, hypercoagulability, or endothelial injury, or develop secondarily on an unstable plaque; such thrombi may carry a higher risk of acute embolization [2,4,17].

By analogy to other territories, mobile/floating atheromatous plaques may contribute to recurrent ischemic events through embolization or progression to occlusion, although evidence in the femoral territory remains limited [6]. In our patient, chronic ischemic complications included delayed wound healing and distal occlusions. While antithrombotic therapy may facilitate resolution of thrombotic masses, it is unlikely to be effective for heavily calcified mobile plaque components, particularly in the CFA [11]. In such settings, surgical removal may offer the most definitive approach and was successfully performed in our case.

While treatment of hemodynamically significant femoral artery stenosis is well established, optimal management of moderately stenotic lesions with a mobile plaque component remains less clearly defined. In the carotid circulation, guideline-based strategies include optimized medical therapy and, in selected cases, intervention [10]. Whether similar principles apply to mobile plaque components in the CFA is uncertain. Broader mechanistic understanding of atherosclerosis continues to evolve, and alternative strategies may warrant further investigation [1].

Raising awareness of this entity and promoting dynamic ultrasound assessment are important, particularly when plaque motion is suspected. Establishing a registry could help clarify incidence, embolic potential, and risk stratification, ultimately guiding future management strategies.

## Conclusions

This case illustrates the value of dynamic B-mode ultrasound for detecting rare mobile plaque components in the CFA and supports femoral endarterectomy as a potential treatment option in this setting. Important questions remain regarding optimal patient selection, the role of medical therapy versus surgery, and whether endovascular or atherectomy-based strategies have a role in this setting.

## References

[1] Poredoš P, Cevc M, Blinc A (2021). Characteristics of atherosclerosis in femoropopliteal artery and its clinical relevance. Atherosclerosis.

[2] Liu W, Lu S, Feng Y, Zhang Z, Liu P, Liu Z (2017). Truth of floating carotid plaques. Front Neurol.

[3] Herisson F, Heymann MF, Chétiveaux M (2011). Carotid and femoral atherosclerotic plaques show different morphology. Atherosclerosis.

[4] Bentzon JF, Otsuka F, Virmani R, Falk E (2014). Mechanisms of plaque formation and rupture. Circ Res.

[5] Delgado MG, Vega P, Roger R, Bogousslavsky J (2011). Floating thrombus as a marker of unstable atheromatous carotid plaque. Ann Vasc Surg.

[6] Fuster V, Moreno PR, Fayad ZA, Corti R, Badimon JJ (2005). Atherothrombosis and high-risk plaque: part I: evolving concepts. J Am Coll Cardiol.

[7] Golemati S, Patelaki E, Gastounioti A, Andreadis I, Liapis CD, Nikita KS (2020). Motion synchronisation patterns of the carotid atheromatous plaque from B-mode ultrasound. Sci Rep.

[8] de Korte CL, Fekkes S, Nederveen AJ, Manniesing R, Hansen HR (2016). Review: mechanical characterization of carotid arteries and atherosclerotic plaques. IEEE Trans Ultrason Ferroelectr Freq Control.

[9] Lin JM, Huang GF, Xie QC, Lyu GR, Lin J, Huang SS, Chen YL (2023). Application of B-flow imaging and its enhanced mode in perforator mapping. Clin Radiol.

[10] Gornik HL, Aronow HD, Goodney PP (2024). 2024 ACC/AHA/AACVPR/APMA/ABC/SCAI/SVM/SVN/SVS/SIR/VESS Guideline for the Management of Lower Extremity Peripheral Artery Disease: a report of the American College of Cardiology/American Heart Association Joint Committee on Clinical Practice Guidelines. J Am Coll Cardiol.

[11] Bonaca MP, Barnes GD, Bauersachs R (2024). Antithrombotic strategies for patients with peripheral artery disease: JACC scientific statement. J Am Coll Cardiol.

[12] Weiss S, Bühlmann R, von Allmen RS, Makaloski V, Carrel TP, Schmidli J, Wyss TR (2016). Management of floating thrombus in the aortic arch. J Thorac Cardiovasc Surg.

[13] Pousios D, Velissaris T, Duggan S, Tsang G (2009). Floating intra-aortic thrombus presenting as distal arterial embolism. Interact Cardiovasc Thorac Surg.

[14] Redgrave JN, Lovett JK, Gallagher PJ, Rothwell PM (2006). Histological assessment of 526 symptomatic carotid plaques in relation to the nature and timing of ischemic symptoms: the Oxford plaque study. Circulation.

[15] Cunnane EM, Mulvihill JJ, Barrett HE, Hennessy MM, Kavanagh EG, Walsh MT (2016). Mechanical properties and composition of carotid and femoral atherosclerotic plaques: a comparative study. J Biomech.

[16] Arbab-Zadeh A, Fuster V (2019). From detecting the vulnerable plaque to managing the vulnerable patient: JACC state-of-the-art review. J Am Coll Cardiol.

[17] Giannopoulos AA, Kyriacou E, Griffin M (2021). Dynamic carotid plaque imaging using ultrasonography. J Vasc Surg.

